# YAP represses intestinal inflammation through epigenetic silencing of JMJD3

**DOI:** 10.1186/s13148-024-01626-w

**Published:** 2024-01-20

**Authors:** Hua Zhu, Jiali Lu, MingYue Fu, Ping Chen, Yali Yu, Min Chen, Qiu Zhao, Min Wu, Mei Ye

**Affiliations:** 1 Department of Gastroenterology, Zhongnan Hospital of Wuhan University, Wuhan, 430071 Hubei China; 2 Hubei Clinical Centre and Key Laboratory of Intestinal and Colorectal Diseases, Zhongnan Hospital, Wuhan University, Wuhan, 430071 Hubei China; 3 Frontier Science Center for Immunology and Metabolism, Hubei Key Laboratory of Cell Homeostasis, Hubei Key Laboratory of Developmentally Originated Disease, Hubei Key Laboratory of Intestinal and Colorectal Diseases, College of Life Sciences, Wuhan University, Wuhan, 430072 Hubei China

**Keywords:** IBD, Hippo pathway, YAP, Histone modification, JMJD3

## Abstract

**Background:**

Epigenetics plays an important role in the pathogenesis of inflammatory bowel disease (IBD). Some studies have reported that YAP is involved in inflammatory response and can regulate target genes through epigenetic modifications. JMJD3, a histone H3K27me3 demethylase, is associated with some inflammatory diseases. In this study, we investigated the role of YAP in the development of IBD and the underlying epigenetic mechanisms.

**Results:**

YAP expression was significantly increased in both in vitro and in vivo colitis models as well as in patients with IBD. Epithelial-specific knockout of YAP aggravates disease progression in dextran sodium sulfate (DSS)-induced murine colitis. In the TNF-α-activated cellular inflammation model, YAP knockdown significantly increased JMJD3 expression. Coimmunoprecipitation experiments showed that YAP and EZH2 bind to each other, and chromatin immunoprecipitation-PCR (ChIP-PCR) assay indicated that silencing of YAP or EZH2 decreases H3K27me3 enrichment on the promoter of JMJD3. Finally, administration of the JMJD3 pharmacological inhibitor GSK-J4 alleviated the progression of DSS-induced murine colitis.

**Conclusion:**

Our findings elucidate an epigenetic mechanism by which YAP inhibits the inflammatory response in colitis through epigenetic silencing of JMJD3 by recruiting EZH2.

**Supplementary Information:**

The online version contains supplementary material available at 10.1186/s13148-024-01626-w.

## Introduction

Inflammatory bowel disease (IBD) is a complex, chronic, and remitting-relapsing inflammatory disorder of the gastrointestinal tract that is sub-classified into Crohn’s disease (CD) and ulcerative colitis (UC) [1, 2]. Although the exact etiology of IBD remains unidentified, recent studies support the idea that it results from a complex interaction between genetic background, environmental factors, intestinal microbiota, and the immune system [3]. UC is a subcategory of IBD, and its two key pathophysiological features are dysregulation of the innate immune system and impaired epithelial regeneration [2]. Treatment options have improved in recent years, but no medication satisfies the demand for UC therapy, and the poor clinical consequences and variable complications remain a challenge for physicians [4–6]. Therefore, studying the mechanisms underlying colitis and identifying new therapeutic targets is an urgent task.

The Hippo pathway, evolutionarily conserved in mammals, is a key regulator of organ size and plays a crucial role in cell proliferation, apoptosis, differentiation, and development [7–9]. It contains a series of kinases, including the core kinase components MST1/2 and LATS1/2, the transcriptional coactivators YAP and TAZ, and the transcriptional factor TEADs (TEAD1-TEAD4) [10]. Among them, YAP is a key transcription coactivator that is negatively regulated through cascade phosphorylation. It is phosphorylated, retained in the cytoplasm, and degraded after ubiquitination. YAP, without phosphorylation, is subsequently transferred into the nucleus, where it interacts with the transcription activator TEAD family and regulates the expression of downstream target genes [7, 10]. Several studies have suggested that YAP is associated with intestinal inflammatory diseases such as IBD [11, 12]. Mounting evidence indicates that the Hippo pathway regulates the function of intestinal epithelial cells, particularly in intestinal epithelial regeneration and tumorigenesis [13–17]. However, its role in IBD pathogenesis remains unclear.

Epigenetic modifications refer to the modulation of gene expression by changing the structure and function of chromatin without altering the DNA sequence, and mainly include DNA methylation, histone modification and non-coding RNA [18]. Mounting evidence indicates that epigenetic regulation is critically involved in inflammatory diseases such as psoriasis, systemic lupus erythematosus and atherosclerosis [19, 20]. With the development of medicine, epigenetic drugs have been increasingly used in clinical practice. Azacitidine, a DNA methyltransferase (DNMT), is the first FDA-approved epigenetically related drug for cancer treatment and is mainly used to treat myelodysplastic syndrome (MDS) [21]. Besides, a variety of epigenetically related drugs have been applied in the clinic for cancer treatment, including Decitabine, Zebularine, Vorinostat, Panobinostat, Chidamide, Belinostat, and Romidepsin etc [20]. Despite abnormal epigenetic modifications in IBD patients affecting the inflammatory phenotype and participating in the development of IBD [22], no FDA-approved drugs are currently available.

Methylation of different lysine residues on histones is important for transcription regulation. Trimethylation of histone H3 on lysine 27 (H3K27me3) is an epigenetic modification associated with transcription inhibition, playing a critical role in tissue development and stem cell fate determination. This modification is catalyzed by methyltransferases, such as EZH2 (enhancer of zeste homolog 2), and removed by demethylases, such as JMJD3 (The Jumonji domain-containing 3, also known as KDM6B) [23]. JMJD3 has been extensively studied in immune diseases, cancer, and tumor development [24–26]. Silencing JMJD3 in synovial fibroblasts increases the enrichment of H3K27me3 on TLR2 promoter, thereby alleviating the severity of rheumatoid arthritis [27]. GSK-J4, a selective inhibitor of the histone demethylase JMJD3, improves experimental autoimmune encephalomyelitis by directly acting on dendritic cells [28]. EZH2 has been implicated in various biological responses, including DNA damage repair, cell senescence, the cell cycle, autophagy, and apoptosis, playing key roles in cellular signaling pathways [29–33]. Inhibition of EZH2 activity alleviated intestinal inflammation in experimental mice and delayed the occurrence of colitis-related cancer [34]. Liu et al. [35] reported that EZH2 could promote inflammation and apoptosis in colitis by integrating various functions of the TNFα signaling pathway. Collectively, these observations suggested the pivotal roles of H3K27me3 in inflammatory diseases.

In this study, we investigated whether YAP is involved in the pathogenesis of intestinal inflammation and its underlying mechanisms. We generated an intestinal epithelium-specific YAP knockout mouse model and found that intestinal deletion of YAP aggravated dextran sodium sulfate (DSS)-induced intestinal inflammation and epithelial damage. Mechanistically, our results highlight an epigenetic mechanism by which YAP inhibits the inflammatory response in colitis through epigenetic silencing of *JMJD3* by binding to EZH2.

## Results

### YAP increased in UC patients and DSS-induced colitis model

**Fig. 1:**
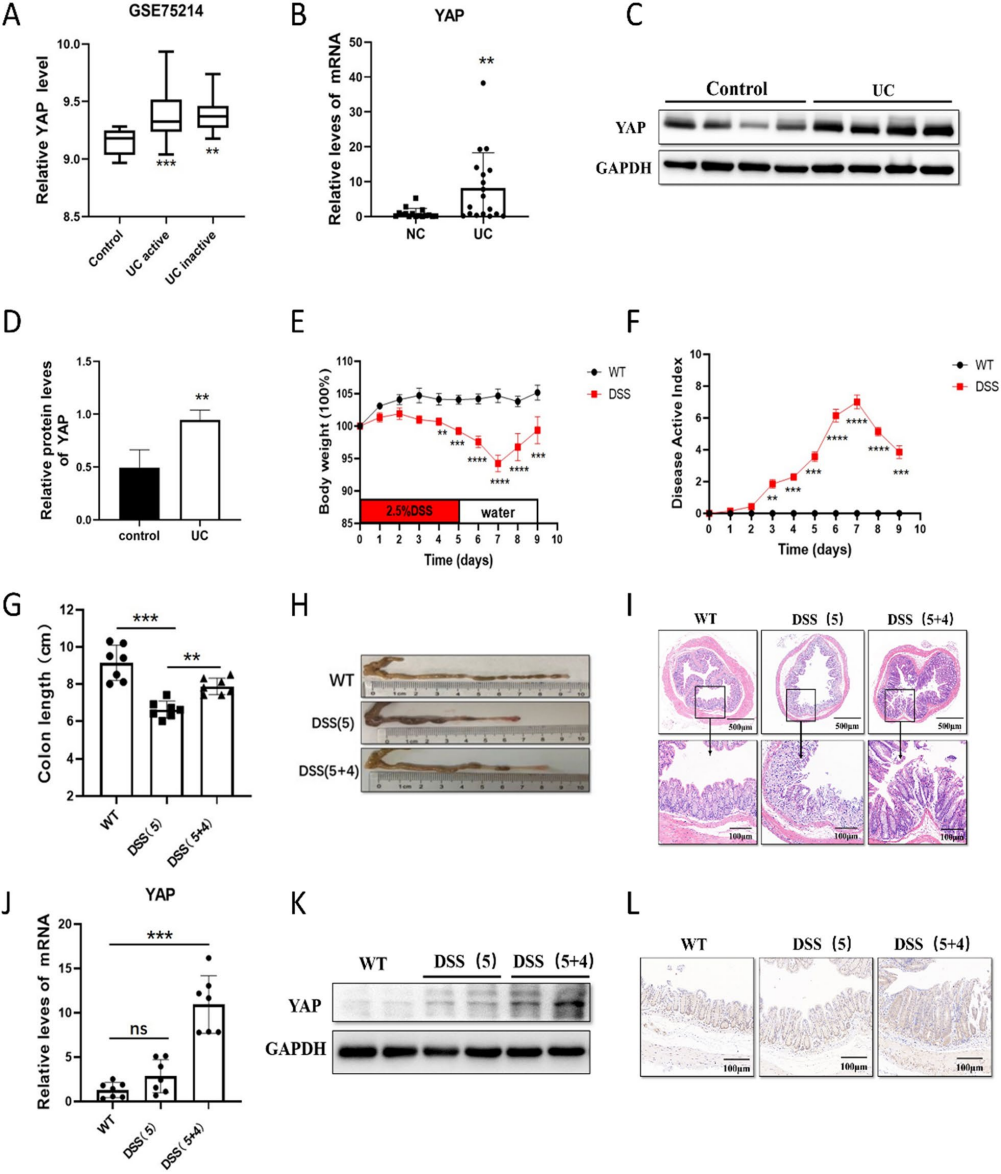
YAP increased in UC patients and DSS-induced colitis model. **A** Boxplot of YAP expression in healthy controls and UC patients (using dataset GSE75214; n = 108). **B** qPCR analysis of YAP in healthy controls (n = 16) and UC patients (n = 17), respectively. **C**–**D** Relative protein levels of YAP in healthy controls and UC patients, respectively. **E**–**H** C57BL/6 mice were fed with 2.5% DSS in drinking water, and loss of body weights (**E**), DAI (**F**) and colon length (**G**, **H**) were recorded. **I** H&E-stained sections of colon tissues collected on days 0, 5 and 9 from 2.5% DSS-treated C57BL/6 mice. **J** qPCR analysis of YAP in NC (n = 7) and 2.5% DSS-induced colitis mice (n = 7). **K** Relative protein levels of YAP in NC and 2.5% DSS-induced acute colitis mice. **L** Immunohistochemical analyses of YAP expressions are shown

To investigate the potential role of YAP in IBD, we analyzed YAP mRNA expression in public datasets of IBD samples (using datasets from NCBI’s Gene Expression Omnibus: GSE75214). The results indicated that YAP mRNA levels were higher in UC specimens than in healthy controls (Fig. 1A). To validate these data, we performed RT-PCR and western blot analyses to detect YAP expression in colonic tissues from patients with UC and normal controls. We found that the YAP level was significantly higher in inflamed colonic mucosa of UC patients compared to that in the normal control (NC) (Fig. 1B–D).

To further evaluate the relevance of YAP in IBD, we established a DSS-induced acute colitis mouse model to simulate the clinical pathology of UC [36]. Compared to the normal control group, mice treated with DSS exhibited lower body weight, higher disease activity index (DAI) score, and shorter colon length. H&E staining confirmed that DSS-treated mice displayed epithelial erosion and inflammation in the colon, indicating the successful establishment of a colitis mouse model (Fig. 1E–I). The results showed that 5 days of 2.5% DSS exposure induced severe intestinal inflammation in the mice. However, after 4 days of DSS withdrawal (5 + 4), the inflammatory changes were largely restored.

Subsequently, we evaluated YAP expression in the intestines of colitis mice. Similar to our observations in UC patients, when compared with the control group, the mRNA and protein levels of YAP in the inflamed tissues of DSS-induced colitis mice did not show significant changes after 5 days of DSS treatment, but obviously increased after 4 days of DSS withdrawal (Fig. 1J–K). Quantification of the immunohistochemical results revealed that in colonic epithelial cells, YAP was significantly higher in the colitis group than in the normal control group (Fig. 1L). These findings suggest a potential link between YAP and intestinal inflammation progress, especially in intestinal epithelial repair and barrier function.

It has been documented that YAP is elevated in colitis models. YAP mRNA levels in intestinal epithelial cells (IECs) are upregulated in the inflamed intestinal tissues of IBD patients and in mice with DSS-induced colitis [16]. The YAP protein level increases in crypts 2 days after DSS withdrawal in DSS-induced colitis [14]. Immunohistochemistry staining indicated that the proportion of elevated YAP expression in UC patient colonic biopsies was significantly higher compared to healthy controls [37]. Elevated YAP promotes the proliferation of the intestinal epithelium and the repair of the intestinal mucosa [14, 16]. In our study, the level of YAP increased after DSS withdrawal, consistent with a previous study.

### YAP^IEC−/−^ mice are more susceptible to DSS-induced colitis

**Fig. 2:**
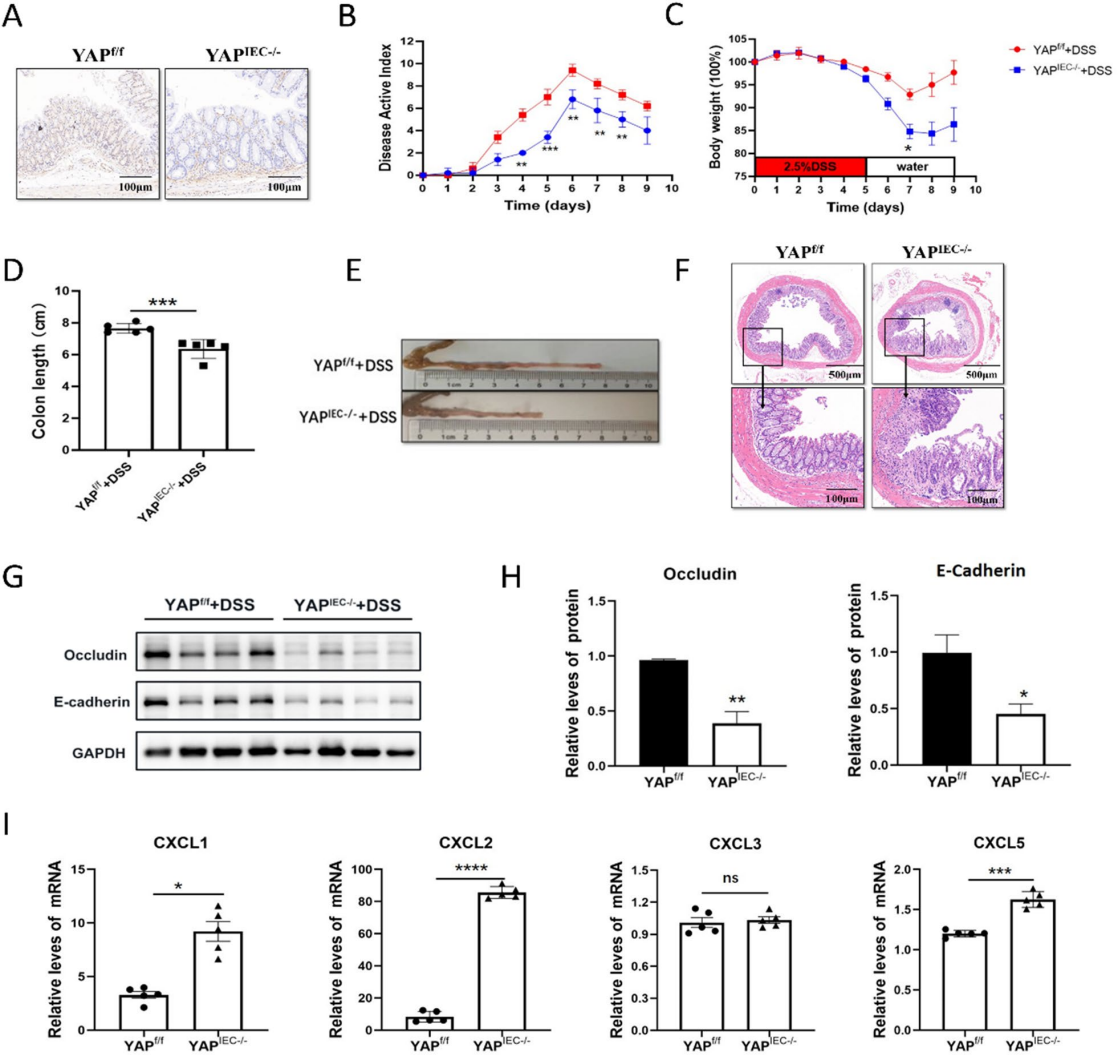
Loss of YAP in IECs aggravates DSS-induced colitis in mice. **A** Immunohistochemical analyses of YAP expressions are shown. **B**–**E** *YAP*^*f/f*^ and *YAP*^*IEC−/−*^ mice were fed with 2.5% DSS in drinking water, and loss of body weights (**B**), DAI (**C**) and colon length (**D**, **E**) were recorded. **F** H&E-stained sections of colon tissues collected on day 9 from 2.5% DSS-treated *YAP*^*f/f*^ and *YAP*^*IEC−/−*^ mice. **G**, **H** Relative protein expression levels of intestinal barrier protein in the whole colon of *YAP*^*f/f*^ and *YAP*^*IEC−/−*^ mice. **I** Relative mRNA expression levels of chemokines in the whole colon of *YAP*^*f/f*^ and *YAP*^*IEC−/−*^ mice

To further define the role of YAP in colonic inflammation, we crossed *YAP*^flox/flox^ mice (*YAP*^f/f^ mice) with *Villin-Cre* mice to obtain an intestinal epithelium-specific YAP knockout mouse strain (*YAP*^IEC−/−^ mice). As expected, immunohistochemical staining revealed that YAP was efficiently ablated in the intestinal epithelium of *YAP*^IEC−/−^ mice (Fig. 2A).

We assessed the consequences of YAP loss in acute colitis by challenging *YAP*^f/f^ and *YAP*^IEC−/−^ mice with 2.5% DSS for 5 days, followed by 4 days of regular drinking water, and then monitored their susceptibility. After DSS administration, *YAP*^IEC−/−^ mice lost more body weight and had higher DAI scores than *YAP*^f/f^ mice (Fig. 2B, C), suggesting that *YAP*^IEC−/−^ mice exhibited more severe intestinal damage and inflammation. Macroscopic dissection revealed significantly shorter colons in *YAP*^IEC−/−^ mice than in *YAP*^f/f^ mice (Fig. 2D–E). Upon further histopathological examination, *YAP*^IEC−/−^ mice exhibited more severe intestinal inflammatory changes, including colonic mucosa structure disorder, crypt damage, and infiltration of inflammatory cells into the mucosal tissue, compared with *YAP*^f/f^ mice (Fig. 2F).

We next explored whether intestinal epithelium-specific YAP knockout affected barrier integrity by assessing the levels of E-cadherin and Occludin in *YAP*^f/f^ and *YAP*^IEC−/−^ mice, which revealed the intercellular junctions of the intestinal mucosa [38]. Reduced protein expression of E-cadherin and Occludin in *YAP*^IEC−/−^ mice compared with control mice indicated that YAP deletion in the colon inhibited intestinal epithelial barrier repair. (Fig. 2G–H).

Since *YAP*^IEC−/−^ mice exhibited higher epithelial damage and inflammation, we analyzed the expression of chemokines in mouse colon tissues, including CXCL1, CXCL2, CXCL3 and CXCL5, which are chemoattractants for innate immune cells and play integral roles in the regulation of the immune response and homeostasis [39]. As expected, the colons of DSS-treated *YAP*^IEC−/−^ mice produced significantly more chemokines than those of DSS-treated *YAP*^f/f^ mice. The intestinal epithelium-specific YAP knockout led to a significant increase in the levels of CXCL1, CXCL2 and CXCL5, whereas the level of CXCL3 remained unaffected (Fig. 2I).

Collectively, these results demonstrated that YAP attenuates the inflammatory response by promoting intestinal epithelial barrier repair and inhibiting the release of chemokines in colitis, suggesting that YAP plays a pivotal role in both intestinal mucosal repair and immune response suppression.

### YAP involved in intestinal inflammation via regulating JMJD3

Although YAP has been reported to play a critical role in the inflammatory process, its underlying mechanism remains unclear. In our previous study [40], we used HCT116 cells to investigate the Hippo pathway and induced YAP activation by FBS treatment after serum starvation. RNA-Seq was used to analyze the gene expression profile, suggesting that JMJD3, a demethylase for H3K27me3, increased after FBS treatment. JMJD3 is associated with immune diseases, cancer, tumor development, and inflammatory responses. It is upregulated in several inflammation diseases and plays a role [41–44]. However, whether JMJD3 is involved in the regulation of YAP-mediated inflammation remains unknown.

**Fig. 3:**
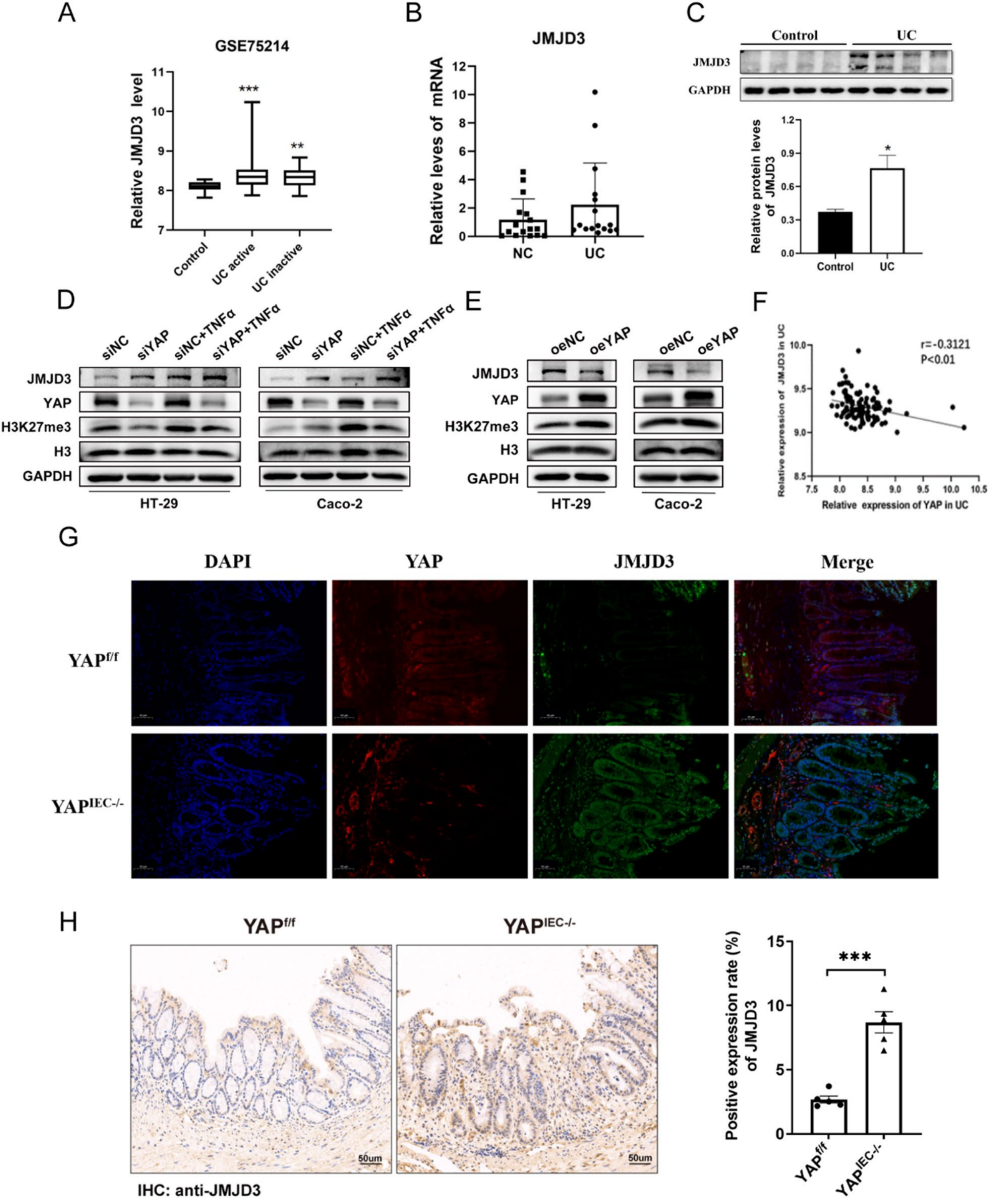
YAP involved in intestinal inflammation via regulating JMJD3. **A** Boxplot of JMJD3 expression in healthy controls and UC patients (using dataset GSE75214; n = 108). **B** qPCR analysis of JMJD3 in healthy controls (n = 16) and UC patients (n = 17), respectively. **C** Relative protein level of JMJD3 in healthy controls and UC patients, respectively. **D** Relative protein expression of YAP, JMJD3 and H3K27me3 in the siNC, siYAP, siNC+ TNF-a, and siYAP+ TNF-a groups across two cell lines. **E** Relative protein expression of YAP, JMJD3 and H3K27me3 in the oeNC and oeYAP groups across two cell lines. **F** The correlation between YAP and JMJD3 in UC patients (using dataset GSE75214; n = 97). **G** Immunofluorescence analysis of intestinal tissue stained with anti-JMJD3 (green) and anti-YAP (red) antibodies; nuclei were stained with DAPI (blue). **H** Representative IHC analysis of JMJD3 expression in colonic tissue samples from DSS-treated *YAP*^f/f^ and *YAP*^IEC−/−^ mice. Quantitative assessment of JMJD3 expression shown on right

Using a public dataset of IBD samples (NCBI’s Gene Expression Omnibus: GSE75214) (Fig. 3A), we found that JMJD3 was upregulated in IBD patients, and our western blot and RT-PCR analyses confirmed this result (Fig. 3B, C). To establish an in vitro inflammation model, HT-29 cells were stimulated with TNF-α at 50 ng/ml for 24 h, while Caco-2 cells were exposed to TNF-α at 100 ng/ml for the same duration. As shown in Additional file 1: Suppl. Fig. 1A, B, RT-PCR showed that the mRNA levels of the inflammatory cytokines and chemokines were significantly increased in response to TNF-α stimulation. To validate whether JMJD3 is a YAP target gene, we examined gene expression in HT29 cells and Caco-2 cells after YAP knockdown and performed western blotting. Surprisingly, we found that YAP deficiency increased JMJD3 protein levels and decreased H3K27me3 protein levels (Fig. 3D). Conversely, both cell lines were transfected with a plasmid expressing constitutively active YAP. As expected, we observed the downregulation of JMJD3 and upregulation of H3K27me3 at the protein level (Fig. 3E). In addition, we analyzed the data in the GEO database GSE75214 and found that JMJD3 expression was negatively correlated with YAP expression in patients with UC (Fig. 3F). Immunofluorescence analysis of intestinal tissues indicated that the numbers of JMJD3 positive cells were significantly higher in colon sections from *YAP*^IEC−/−^ mice than in those from *YAP*^f/f^ mice (Fig. 3G). Similarly, we conducted immunohistochemical analysis of JMJD3 in colon sections of transgenic mice after DSS-induced colitis. The results indicated that JMJD3 expression was significantly higher in *YAP*^IEC−/−^ mice compared to *YAP*^f/f^ mice (Fig. 3H).

Overall, our results demonstrate that JMJD3 is a target of YAP, and this work demonstrates an aspect of YAP nuclear function as a transcriptional repressor. However, the mechanisms through which YAP regulates JMJD3 remain unclear.

### EZH2 is required for YAP-mediated repression of JMJD3

**Fig. 4:**
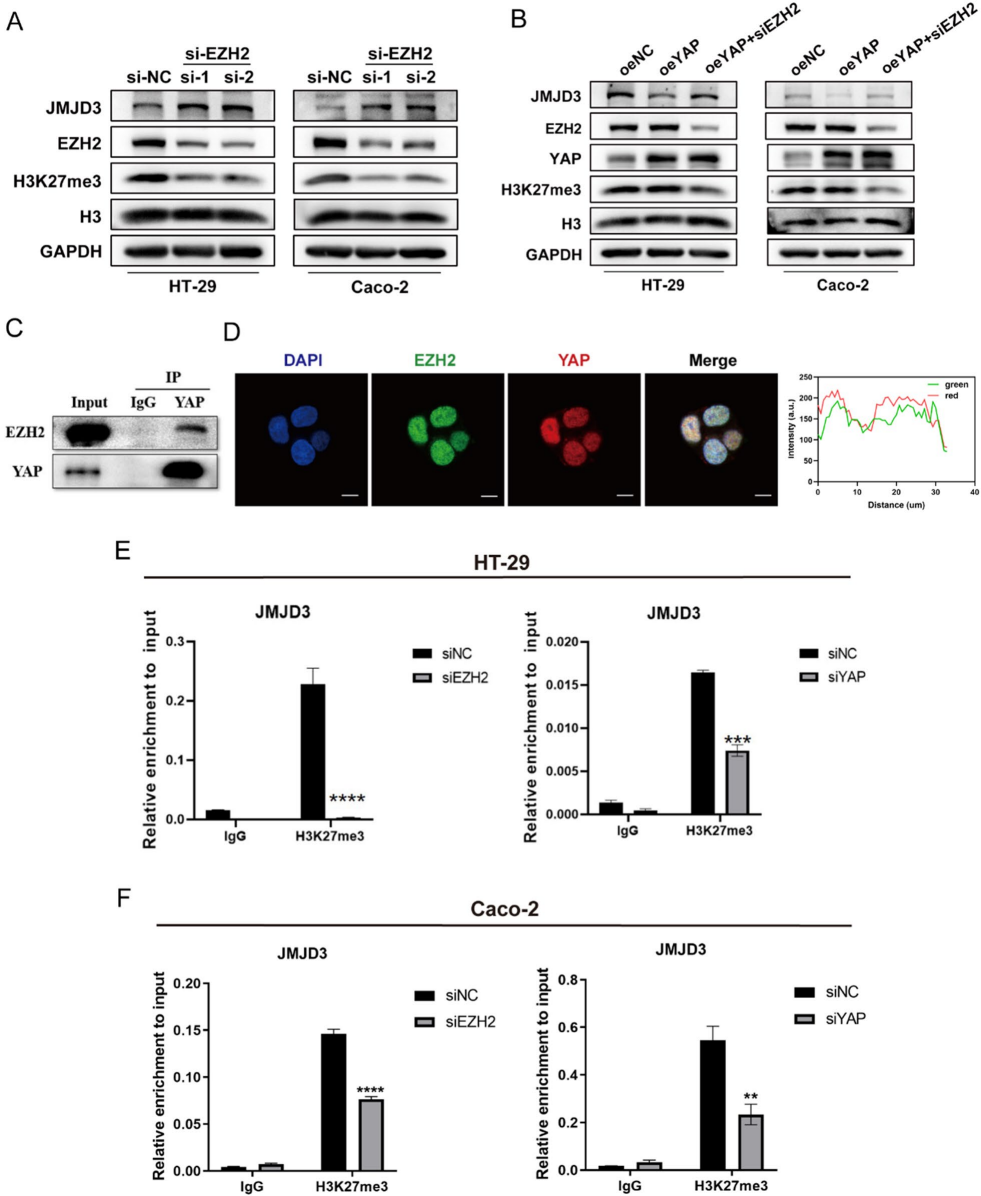
EZH2 is required for YAP-mediated repression of JMJD3. **A** Relative protein expression of EZH2, JMJD3 and H3K27me3 after transfection with siEZH2-1,2, or negative control in two cell lines. **B** Relative protein expression of YAP, EZH2, JMJD3 and H3K27me3 in the oeNC, oeYAP, and oeYAP+ siEZH2 groups across two cell lines. **C** Co-IP experiment in HT29 cells was performed to validate the combination between YAP and EZH2. **D** Immunofluorescence colocalization analysis of Caco-2 cells stained with anti-EZH2 (green) and anti-YAP (red) antibodies; nuclei were counterstained with DAPI (blue). Quantification of immunofluorescence data shown on right. **E**, **F** ChIP-PCR analysis to validate the enrichment of H3K27me3 on the JMJD3 promoter in **E** HT29 cells and **F** Caco-2 cells transfected with siEZH2, siYAP or siNC, with IgG used as a negative control

In addition to its well-known function as a transcriptional activator, YAP functions as a transcriptional repressor by interacting with the polycomb repressive complex member EZH2 in cancer [45–47]. We then explored whether YAP downregulated JMJD3 by interacting with EZH2 during inflammation. We knocked down EZH2 using siRNA in both cell lines and observed upregulation of JMJD3 and downregulation of H3K27me3 at the protein levels (Fig. 4A), suggesting that, in addition to YAP, EZH2 also represses JMJD3 expression.

Given that both YAP and EZH2 suppress JMJD3, and considering a previous study suggesting that YAP functions as a transcriptional repressor by interacting with EZH2, we explored whether YAP represses JMJD3 by interacting with EZH2. We exogenously expressed YAP in two cell lines, followed by EZH2 transient knockdown using siRNA, and found that YAP overexpression failed to repress JMJD3 protein expression when simultaneously silencing EZH2 (Fig. 4B). Immunoprecipitation results in HT29 cells showed that endogenous YAP binds to EZH2 (Fig. 4C). Immunofluorescence co-localization staining of Caco-2 cells confirmed the co-localization of YAP and EZH2 in the nuclei (Fig. 4D). The above results collectively indicate that YAP functions as a transcriptional repressor by interacting with EZH2, which is consistent with previous reports.

To clarify the mechanism underlying YAP regulation of JMJD3, we performed chromatin immunoprecipitation (ChIP)-PCR experiments. Since EZH2 represses gene expression through trimethylation of H3K27, we employed a specific antibody against H3K27me3 for ChIP-PCR analysis in two cell lines and observed enrichment of the JMJD3 promoter. Furthermore, H3K27me3 enrichment on the JMJD3 promoter was significantly decreased in the siEZH2 and siYAP groups compared to that in the siNC group (Fig. 4E, F).

Overall, our results demonstrate that YAP suppresses JMJD3 by interacting with EZH2, thereby regulating H3K27me3 on the promoter of JMJD3.

### JMJD3-specific inhibitor GSK-J4 alleviated DSS-induced colitis in wild-type (WT) and *YAP*^IEC−/−^ mice

**Fig. 5:**
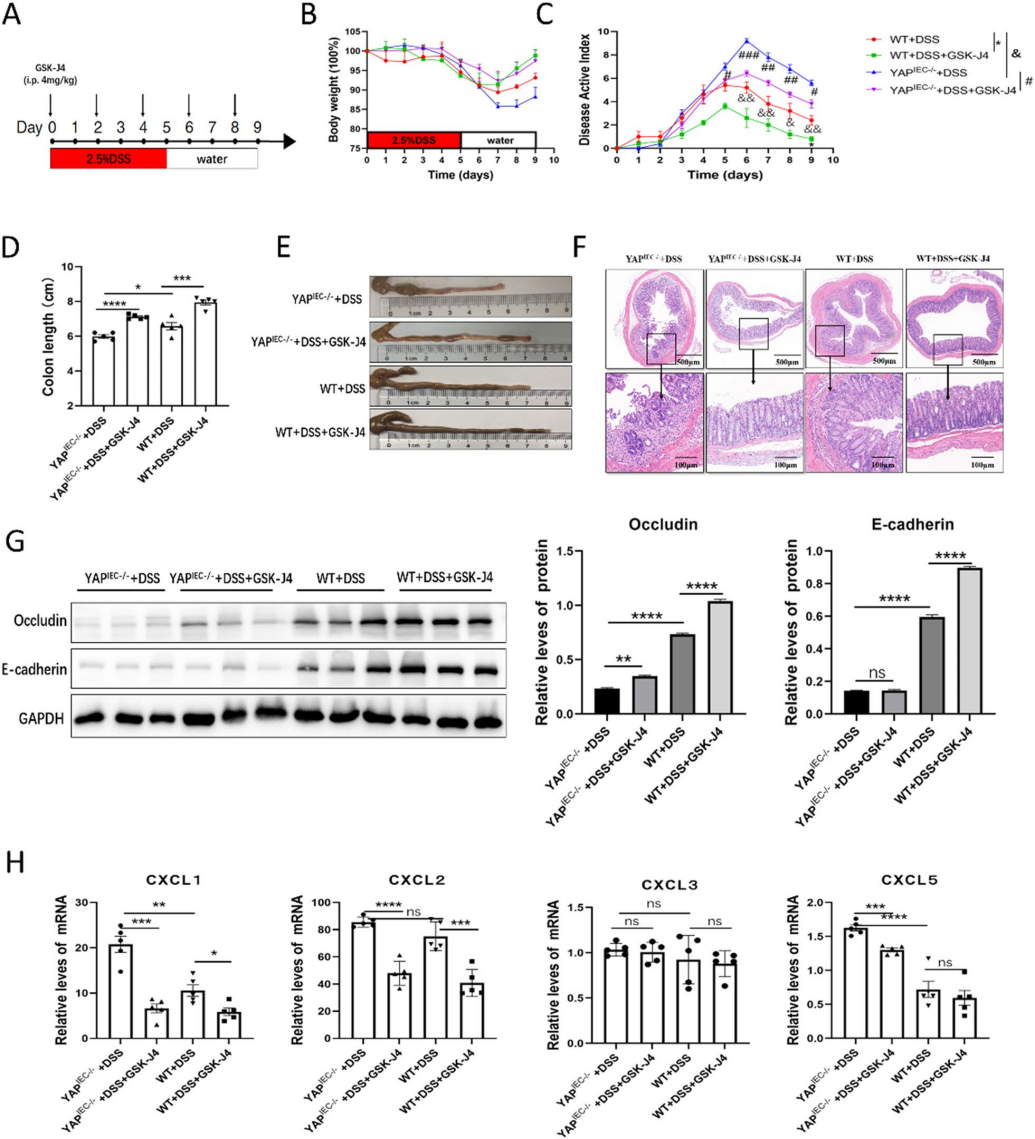
JMJD3 inhibitor GSK-J4 alleviate DSS-induced mice colitis. **A** Schematic representation of the DSS protocol used to induce acute colitis in *YAP*^*IEC−/−*^ mice, along with GSK-J4 administration. **B**–**E** Body weight (**B**), DAI (**C**), and colon length (**D**, **E**) of mice in the WT, WT + GSK-J4, *YAP*^*IEC−/−*^ and *YAP*^*IEC−/−*^+ GSK-J4 groups. (**F**) H&E-stained sections of colon tissues collected from the WT, WT+ GSK-J4, *YAP*^*IEC−/−*^ and *YAP*^*IEC−/−*^+ GSK-J4 groups. **G** Relative protein expression levels of intestinal barrier protein in the whole colon of the WT, WT+ GSK-J4, *YAP*^*IEC−/−*^ and *YAP*^*IEC−/−*^+ GSK-J4 groups. **H** Relative mRNA expression levels of chemokines in the whole colon of the WT, WT+ GSK-J4, *YAP*^*IEC−/−*^ and *YAP*^*IEC−/−*^+ GSK-J4 groups

To further investigate the effect of the YAP-EZH2-JMJD3 regulatory axis in vivo, we used a mouse model of DSS-induced acute colitis. C57BL/6 WT and *YAP*^IEC−/−^mice were intraperitoneally injected with GSK-J4 or a placebo every other day (Fig. 5A). After DSS administration, *YAP*^IEC−/−^ mice exhibited more significant body weight, higher DAI scores, shorter colons, and more severe intestinal inflammatory changes than WT mice with or without GSK-J4 treatment, which is consistent with previous results (Fig. 5B–F). In addition, compared with the DSS-treated group, mice treated with DSS + GSK-J4 experienced reduced body weight loss and lower DAI score (Fig. 5B, C), manifested significantly longer colons upon macroscopic dissection (Fig. 5D, E), and displayed less intestinal inflammation as indicated by H&E staining (Fig. 5F). These results suggest that GSK-J4 alleviates inflammation and intestinal damage in both WT and *YAP*^IEC−/−^ mice.

In addition, western blotting revealed that the expression of Occludin and E-cadherin in *YAP*^IEC−/−^ mice was lower than that in WT mice, regardless of GSK-J4 treatment, and notably, the DSS + GSK-J4 group exhibited increased expression compared to the DSS-treated group (Fig. 5G). These results suggest that JMJD3 inhibition promotes intestinal epithelial barrier repair in both WT and *YAP*^IEC−/−^ mice. Next, we analyzed the expression of chemokines. Similarly, intestinal epithelium-specific YAP knockout led to a significant increase in the levels of CXCL1, CXCL2 and CXCL5 with or without GSK-J4, and GSK-J4 administration led to a significant decrease in CXCL1, CXCL2 and CXCL5 *i*n both WT and *YAP*^IEC−/−^ mice, whereas the level of CXCL3 remained unaffected (Fig. 5H).

Taken together, YAP deletion in the colon aggravated colitis and JMJD3 inhibition alleviated DSS-induced acute colitis in both WT and *YAP*^IEC−/−^ mice by promoting barrier integrity and downregulating chemokines.

## Discussion

**Fig. 6:**
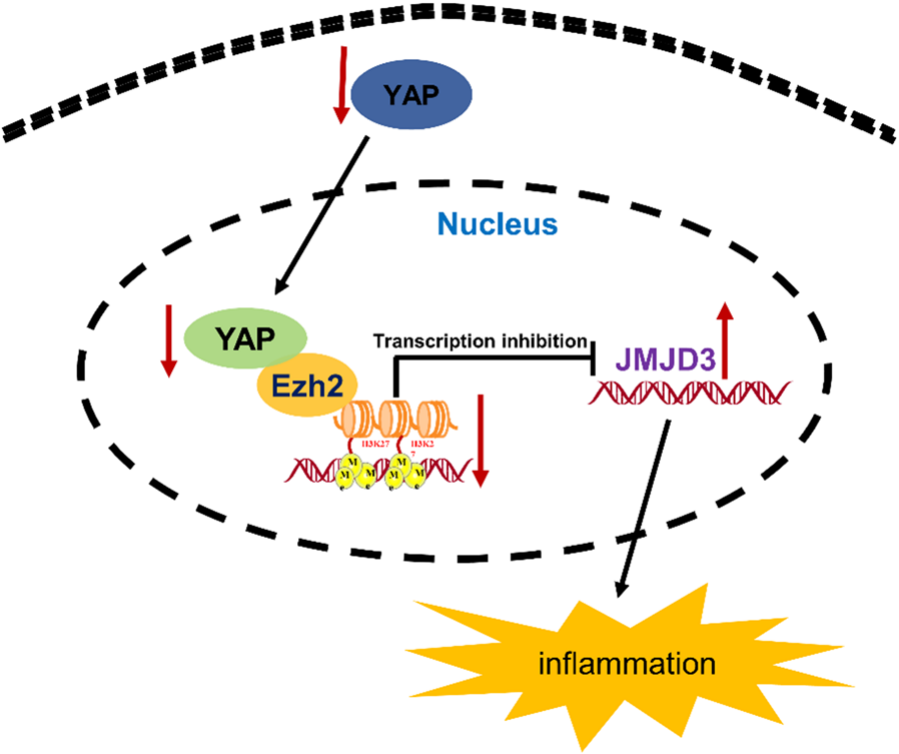
Schematic illustrating the molecular mechanisms underlying YAP to regulate JMJD3 in the inflammatory pathogenesis of colitis

The Hippo pathway is a classical pathway involved in tumor signal transduction [48]. Previous studies have shown that it controls organ size and tumor development by regulating cell proliferation, apoptosis, and stem cell growth [7–9]. Although it has been well studied that YAP acts as a transcriptional coactivator in the gut, many important questions remain unclear. In the current study, we found that YAP was upregulated in the colonic tissues of patients with UC and DSS-induced colitis in mice. In addition, epithelial-specific knockout of the YAP gene aggravated disease progression. We identified that YAP inhibits the colitis inflammatory response and promotes intestinal epithelial barrier repair through epigenetic silencing of JMJD3 by binding to EZH2. We conclude that YAP is an important regulator of intestinal inflammation (Fig. 6).

YAP functions as a transcriptional coactivator in the Hippo pathway and plays critical roles in intestinal diseases, especially in the regeneration of intestinal epithelial cells [49]. In a DSS-induced colitis model, activation of YAP accelerated the proliferation of colonic epithelial cells within crypts, promoting intestinal tissue repair and colon regeneration through extracellular matrix (ECM) remodeling [50]. MicroRNA-31 promotes epithelial regeneration after intestinal injury by regulating the WNT and Hippo signaling pathways [51]. Moreover, PGE2 signal transduction can promote colon tissue regeneration and significantly improve the healing ability of IECs in colitis mice by increasing the expression and transcriptional activity of YAP [52]. However, some studies have reported that YAP promotes an inflammatory response by activating the NLRP3 inflammasome in macrophages, controlling M1/M2 macrophage polarization, and altering intestinal microbial homeostasis [53, 54]. In our investigation using the DSS-induced colitis model, we found that YAP expression was markedly enhanced 4 days after DSS removal, consistent with a previous finding [55]. In that study, within the murine DSS-induced colitis and repair model, the expression of YAP in the colonic mucosa exhibits temporal modulation and fluctuation, with YAP expression being enhanced during epithelial regeneration following DSS-induced colitis in mice [55]. However, the expression changes of YAP in UC patients showed inconsistency. In our study, we observed an increase in YAP expression compared to healthy controls. This observation may be associated with distinct stages in the progression of inflammatory injury and repair in the colonic mucosa collected from UC patients in our investigation. Notably, a study conducted immunohistochemistry on the colonic epithelium from healthy control and IBD patient biopsies for YAP1, revealing that the proportion of elevated YAP expression in UC patient colonic biopsies was significantly higher compared to healthy controls [37]. In conclusion, the expression of YAP in UC patients is closely associated with the inflammatory and wound healing stages of the disease. Moreover, in vivo inflammation model, depletion of YAP led to exacerbated colitis, suggesting that YAP plays a pivotal role in promoting epithelial regeneration and tissue repair to enhance the intestinal barrier and attenuate inflammatory responses in experimental colitis.

Damage to the epithelial barrier is a major factor in intestinal inflammatory disease, which contributes to intestinal inflammation and mucosal damage by reducing the expression of intestinal barrier proteins at intercellular junctions [56]. Previous studies have shown that declining levels of E-cadherin and Occludin are significantly associated with the intensity of colitis [57, 58]. In this study, reduced protein expression of E-cadherin and Occludin in *YAP*^IEC−/−^ mice indicated that YAP deletion in the colon inhibits intestinal epithelial repair. Furthermore, we found that intestinal epithelium-specific YAP knockout led to a significant increase in CXCL1, CXCL2 and CXCL5. These results indicate that *YAP*^IEC−/−^ mice are more susceptible to DSS-induced colitis.

In the current study, we identified JMJD3 as a YAP target gene. Existing evidence suggests that JMJD3 plays a key role in inflammatory and autoimmune diseases. Silencing of JMJD3 has been shown to improve the severity of rheumatoid arthritis (RA) in synovial fibroblasts and ameliorate disease severity in experimental autoimmune encephalomyelitis [27, 28]. JMJD3 depletion negated downstream NF-κB proinflammatory signaling and subsequently reduced the LPS-stimulated upregulation of TNF-α, IL-1β, and IL-6 [44]. Furthermore, JMJD3 inhibition improved necrotizing enterocolitis (NEC) by attenuating the inflammatory response and ameliorating intestinal injury via the NF-κB and JAK2/STAT3 pathways in NEC mice [59]. Remarkably, targeting JMJD3 not only holds therapeutic potential for the treatment of inflammatory diseases but also represents a potential target for the treatment of IBD. Consistent with these studies, we found that the expression of JMJD3 was increased in both the inflammatory cell model and the intestinal mucosa of UC patients. Silencing or overexpression of YAP affects the expression of JMJD3 in the inflammatory microenvironment of HT29 and Caco-2 cells. In addition, using the GEO database, we found that JMJD3 expression was negatively correlated with the expression of YAP in patients with UC. Furthermore, the number of JMJD3 positive cells was significantly higher in the *YAP*^IEC−/−^ mice. These results show that YAP inhibits inflammation and promotes intestinal epithelial repair at least partly through the regulation of JMJD3.

To further illustrate the regulation between YAP and JMJD3, we investigated the underlying mechanism. YAP has been implicated as a transcriptional coactivator of oncogenes in various cancers [60–63]. In addition to this, YAP functions as a transcriptional repressor by interacting with EZH2. YAP and EZH2 act cooperatively to impair the tumor suppressor activity of TGFBR2 in non-small cell lung cancer [46], and the interaction between YAP and EZH2 participates in the transcriptional repression of key cell cycle regulators [45]. Moreover, YAP represses GDF15 transcription to promote metastasis of breast cancer cells by recruiting EZH2 and trimethylating histone H3 lysine 27 on the promoter of GDF15 [47]. In our study, coimmunoprecipitation and immunofluorescence co-localization experiments demonstrated that YAP and EZH2 physically interacted with each other. Furthermore, the ChIP experiment verified that silencing YAP or EZH2 significantly decreased H3K27me3 enrichment on JMJD3. Therefore, we have demonstrated that YAP downregulates JMJD3 by interacting with EZH2 in inflamed tissues.

Finally, we demonstrated that JMJD3 inhibition by GSK-J4 administration alleviated DSS-induced acute colitis in both WT and *YAP*^IEC−/−^ mice. Previous studies have shown that JMJD3 serves as an inflammatory factor in a mouse model of colitis. Treatment with a JMJD3 inhibitor significantly alleviated intestinal inflammation and decreased inflammatory factor expression in the mouse colon, implicating an inflammatory role of JMJD3 in IBD [28, 64, 65]. GSK-J4 can reduce inflammatory enteritis by diminishing the proinflammatory potential of dendritic cells and attenuate the progression of DSS-induced colitis in mice in vivo, including reducing the disease activity index, improving body weight, and preventing intestinal shortening [64, 65]. In the current study, we found that YAP deletion in the colon aggravated colitis. However, GSK-J4 alleviated DSS-induced and *YAP*^IEC−/−^-aggravated acute colitis by suppressing the proinflammatory response with reduced levels of chemokines and ameliorating intestinal injury by reversing Occludin and E-cadherin expression. Our study verified the effects of the YAP-JMJD3 regulatory axis in vivo. Thus, pharmacological agonists or mimics of YAP and JMJD3-specific inhibitors could be developed as potential medications for IBD.

Limitations of our study include that—we did not examine the expression of YAP at different disease stages, considering the inconsistency in YAP expression in UC. In the DSS-induced colitis model, it would be beneficial to sample at multiple time points to analyze YAP expression during both the inflammatory and repair stages. Additionally, evaluation YAP expression in the large-scale sampling of UC patients will contribute to a more comprehensive and accurate understanding of YAP expression patterns in different pathological states of UC. Furthermore, although JMJD3 has been identified as a proinflammatory factor in mouse colitis models [28, 65], we have not validated the downstream pathways through which JMJD3 promotes colitis in our study. In our future research endeavors, we are committed to addressing this aspect to enhance the depth and completeness of our findings.

## Conclusion

YAP is an important regulator of intestinal inflammation, and its underlying mechanism is partly attributed to the regulation of JMJD3 expression. We demonstrated that YAP represses JMJD3, which is a novel target gene of YAP, by engaging the EZH2 epigenetic repressor and promoting H3K27me3.

## Materials and methods

### Specimen collection

A total of 17 patients with active UC were recruited at Zhongnan Hospital of Wuhan University (Wuhan, China) between May 2021 and September 2021. All patients were diagnosed based on clinical and endoscopic features, original histopathological detection, and imaging appearance. The healthy controls (n = 16) were age- and sex-matched healthy volunteers. Inflamed UC tissues collected during the endoscopic procedure or operation were preserved in liquid nitrogen. The study was approved by the Clinical Research Institutional Review Committee and Ethics Review Committee of Zhongnan Hospital (Scientific Ethical Approval No. [2019020]).

### Animals

*YAP*^flox/flox^ mice were kindly gifted by Prof. Zhang Lei from the Shanghai Institute of Biochemistry, Chinese Academy of Sciences. *Villin-Cre* mice were kindly gifted by Prof. Liu Zhanju from Tongji University, China. *YAP*^IEC−/−^ mice were generated by crossing *YAP*^flox/flox^ mice with *Villin-Cre* mice. All mice were maintained on a C57BL/6 background. WT C57BL/6 mice were obtained from the Institute of Model Animals, Wuhan University. All mice were bred under specific pathogen-free conditions, with a one-week acclimatization period prior to the experiments. All animal experimental procedures were approved by the Committee on the Ethics of Animal Experiments at Zhongnan Hospital, Wuhan University.

### Induction of colitis model and drug application

To induce acute colitis, mice were fed with 2.5% DSS (MP Biomedicals) in their drinking water for 5 days, followed by 4 days of regular water. For GSK-J4 (MedChemExpress, USA) treatment experiments, GSK-J4 was dissolved in dimethyl sulfoxide (DMSO, Sigma, USA) and further diluted in corn oil for in vivo studies. Mice were treated with GSK-J4 intraperitoneally every other day at a dose volume of 4 mg/kg body weight. Equivalent volumes of DMSO and corn oil were injected into the vehicle control mice. The treatment started on day 0 and continued until the mice were sacrificed. The DAI score was used to quantify the severity of colitis, as previously described [66] and shown in Table 1).

**Table 1:** Disease activity index scoring system

Score	Weight loss (%)	Stool consistency	Occult/gross bleeding
0	None	Normal	Normal
1	1–5	–	–
2	6–10	Loose stools	Hemoccult positive
3	11–20	–	–
4	> 20	Diarrhea	Gross bleeding

### Cell culture and stimulation

The human colon carcinoma cell lines, HT29 and Caco-2, were purchased from the China Center for Type Culture Collection (Wuhan, China). All cells were cultured at 37 °C with 5% CO_2_. The HT29 cell line was maintained in RPMI medium (HyClone, USA) containing 10% fetal bovine serum (FBS, Gibco, USA) and 100 ul/ml of Penicillin–Streptomycin Solution (Genom, China). The Caco-2 cell line was maintained in MEMα medium (Pricella, China) with 20% FBS (Gibco, USA) and 100 ul/ml of Penicillin–Streptomycin Solution (Genom, China). For the inflammatory cell model, cells were grown to 70% confluency, and HT-29 cells were then stimulated with TNF-α (PeproTech, USA) at 50 ng/ml for 24 h, while Caco-2 cells were exposed to TNF-α at 100 ng/ml for the same duration.

### Cell transfection with small interfering RNA

YAP, EZH2, and control small interfering RNA (siRNA) (Guangzhou RiboBio, China) were transfected into HT29 and Caco-2 cells using Lipofectamine 2000 (Invitrogen, USA) according to the manufacturer’s instructions. The efficiency of siRNA transfection was further validated by qRT-PCR and Western blotting. Experiments were performed 48–72 h after transfection.

### RNA extraction and quantitative RT-PCR analysis

Total RNA from colonic tissues or cell lines was extracted using TRIzol reagent (Invitrogen, USA) or the EASY spin Tissue/Cell RNA Rapid Extraction Kit (Aidlab, China) according to the manufacturer’s instructions. cDNA was synthesized from each RNA sample using the TOYOBO ReverTra Ace kit (TOYOBO, Japan). mRNA expression was quantified using a LightCycler96 (Roche, USA). The mRNA expression levels were calculated using the 2ΔΔCt quantification method, with GAPDH as the housekeeping gene. All experiments were performed in triplicate. The primers were designed using the Primer Bank website [67] and synthesized by TSINGKE Biological Technology (Wuhan, China). All primer sequences are listed in Table 2.

**Table 2:** Primer sequences

Gene	Species	Forward primer	Reverse primer
GAPDH	Homo	5′GGAGCGAGATCCCTCCAAAAT 3′	5′GGCTGTTGTCATACTTCTCATGG 3′
YAP	Homo	5′TAGCCCTGCGTAGCCAGTTA 3′	5′TCATGCTTAGTCCACTGTCTGT 3′
JMJD3	Homo	5′CGCTGCCTCACCCATATCC 3′	5′ATCCGCGACCTCTGAACTCT 3′
EZH2	Homo	5′AATCAGAGTACATGCGACTGAGA 3′	5′GCTGTATCCTTCGCTGTTTCC 3′
IL-1β	Homo	5′ATGATGGCTTATTACAGTGGCAA3′	5′GTCGGAGATTCGTAGCTGGA 3′
IL-8	Homo	5′ACTGAGAGTGATTGAGAGTGGAC 3′	5′AACCCTCTGCACCCAGTTTTC 3′
IL-12	Homo	5′AGCTTTGCATTCATGGTCTTGA 3′	5′ATGGCCCTGTGCCTTAGTAGT 3′
IL-18	Homo	5′TCTTCATTGACCAAGGAAATCGG 3′	5′TCCGGGGTGCATTATCTCTAC 3′
CXCL3	Homo	5′CGCCCAAACCGAAGTCATAG 3′	5′GCTCCCCTTGTTCAGTATCTTTT 3′
CXCL2	Homo	5′CTGCGCTGCCAGTGCTT3′	5′CCTTCACACTTTGGATGTTCTTGA3′
CXCL5	Homo	5′AGCTGCGTTGCGTTTGTTTAC3′	5′TGGCGAACACTTGCAGATTAC3′
TNF	Homo	5′GAGGCCAAGCCCTGGTATG3′	5′CGGGCCGATTGATCTCAGC3′
COX2	Homo	5′GTCTGATGATGTATGCCACAATCTG3′	5′GATGCCAGTGATAGAGGGTGTTAAA3′
INOS	Homo	5′AGGGACAAGCCTACCCCTC3′	5′CTCATCTCCCGTCAGTTGGT3′
NF-κB	Homo	5′AACAGAGAGGATTTCGTTTCCG3′	5′TTTGACCTGAGGGTAAGACTTCT3′
GAPDH	Mus	5 ‘AGGTCGGTGTGAACGGATTTG3′	5′TGTAGACCATGTAGTTGAGGTCA3′
YAP	Mus	5′TGTTGTTGTCTGATCGTTGTGAT 3′	5′TGAGATCCCTGATGATGTACCAC 3′
JMJD3	Mus	5′TGAAGAACGTCAAGTCCATTGTG 3′	5′TCCCGCTGTACCTGACAGT3′
CXCL1	Mus	5′CCACACTCAAGAATGGTCGC	5′TCTCCGTTACTTGGGGACAC
CXCL2	Mus	5′GAGCTTGAGTGTGACGCCCCCAGG	5′GTTAGCCTTGCCTTTGTTCAGTATC
CXCL3	Mus	5′CCTACCAAGGGTTGATTTTGAGAC	5′AGTGGCTATGACTTCTGTCTGGGT
CXCL5	Mus	5′GTTCCATCTCGCCATTCATGC	5′GCGGCTATGACTGAGGAAGG

### Protein extraction and western blotting

Proteins in the cells and colonic tissues were extracted using RIPA lysis buffer (Boyotime, China) supplemented with 1% phenylmethylsulfonyl fluoride (PMSF) (Beyotime, China). The BCA kit (Beyotime, China) was used to determine protein concentrations. Western blotting was performed with specific antibodies, including anti-YAP (1:1000, Cell Signaling Technology, 14074), anti-JMJD3 (1:1000, Cell Signaling Technology, 3457), anti-EZH2 (1:1000, Cell Signaling Technology,5246), anti-E-cadherin (1:1000, Cell Signaling Technology, 14472), anti- H3K27me3 (1:1000, Cell Signaling Technology,9733), anti-GAPDH (1:3000, Proteintech, 60004-1-Ig), anti-H3(1:1000, Proteintech, 17168-1-AP), and anti-Occludin (1:1000, Proteintech, 27260-1-AP). Images were visualized using the GeneSys software (Thermo, USA). Gray analysis of the Western blot results was performed using ImageJ software.

### Immunofluorescence staining of colonic tissue

The Paraffin-embedded colonic tissue sections were deparaffinized, blocked with 5% bovine serum albumin (BSA) for 1 h, and then incubated with primary antibodies at 4 °C overnight (anti-JMJD3, 1:50, Abclonal, A17382; anti-YAP, 1:50, Cell Signaling Technology, 14,074). After washing with PBS, the sections were incubated with secondary antibodies (Thermo Fisher Scientific) at 37 °C for 2 h in the dark. Nuclei were counterstained with DAPI (Antgene). All images were obtained using a confocal microscope (Olympus, Tokyo, Japan).

### Immunofluorescence co-localization staining of Caco-2 cells

Caco-2 cells grown on the confocal dish were fixed with 4% paraformaldehyde for 15 min and permeabilized by exposure to 0.5% Triton X-100 in PBS for 15 min. After blocking with 5% bovine serum albumin (BSA) for 1 h at room temperature, the cells were incubated with primary antibodies at 4 ׄ°C overnight. The primary antibodies used were as follows: YAP Rabbit mAb (Cat#:14074, 1:100, CST), Ezh2 Mouse mAb (Cat#:3147, 1:100, CST). After thorough washing with PBS containing 0.1% Tween20, these cells were incubated with Cy3-labeled goat anti-rabbit IgG (Servicebio, China) and DyLight 488-conjugated goat anti-mouse IgG (Abbkine, China) in the dark for 1 h at room temperature. Nuclei were visualized through DAPI staining. Immunofluorescence images were obtained using a confocal microscope (Leica STELLARIS 5 SR, Germany).

### Chromatin immunoprecipitation-PCR assay

HT29 and Caco-2 cells were fixed in 1% formaldehyde and incubated for 10 min at room temperature. Glycine was then added to stop crosslinking, and cells were incubated for an additional 5 min at room temperature. Lysis buffer containing protease inhibitors (MCE, USA) was added to suspend the cells, and the cell lysates were sonicated using EPISONIC (USA) to obtain chromatin fragments ranging from 200 to 1000 bp. Immunoprecipitation was performed using H3K27me3 (1:50, Cell Signaling Technology, 9733)-specific antibodies and IgG (1:100, Cell Signaling Technology, 2729S). DNA was extracted using a DNA purification kit (TIANGEN, China), and specific primers for the JMJD3 promoter were used for PCR. The primer sequences were as follows: JMJD3: F 5′-GTGAGGGATTAAAGCTCTGGGG-3′; R 5′-GAGTGGGCAGACTTTAGGACA-3′.

### Coimmunoprecipitation

Cell lysates were prepared using NP40 lysis buffer (Beyotime, China) supplemented with 1% PMSF (Beyotime, China). After centrifugation at 15,000 rpm for 20 min at 4 °C, the supernatant was equally distributed into two new 1.5 ml tubes. YAP antibody (1:50, Cell Signaling Technology, 14,074) and IgG (1:100, Cell Signaling Technology, 2729S) were added, and the tubes were gently swirled and mixed overnight at 4 °C. After incubation overnight, 20 µL of Protein A Magnetic Beads (Cell Signaling Technology, 73778) were added to each tube and mixed gently at 4 °C for 2–4 h. Magnetic Beads were separated using a magnetic frame and washed five times with lysis buffer. SDS loading buffer was then added to the beads, followed by western blotting.

### Data analysis

All experiments were independently performed three times. Data are presented as mean ± SD. Statistical analysis was conducted using GraphPad Prism software (version 9.0; GraphPad Software, USA). Statistical significance between different groups was determined using one-way analysis of variance (ANOVA). The least significant difference (LSD) t-test was used for multiple comparisons within the same group. Statistical significance was defined as *P* < 0.05.

### Supplementary Information

**Additional file 1. Suppl. Fig. 1:** Inflammatory cytokines were increased in vitro inflammatory model.

## Data Availability

The data underlying this article will be shared upon reasonable request with the corresponding author.
