# Supplementary material for: YAP represses intestinal inflammation through epigenetic silencing of JMJD3
Source: Clin Epigenetics. 2024 Jan 20;16:14. doi: 10.1186/s13148-024-01626-w (PMC10800074; doi:10.1186/s13148-024-01626-w)
Supplement: Supplementary file 1 — Additional file 1. Suppl. Fig. 1: Inflammatory cytokines were increased in vitro inflammatory model. [file 13148_2024_1626_MOESM1_ESM.docx]

**Supplementary figures and figure legends**


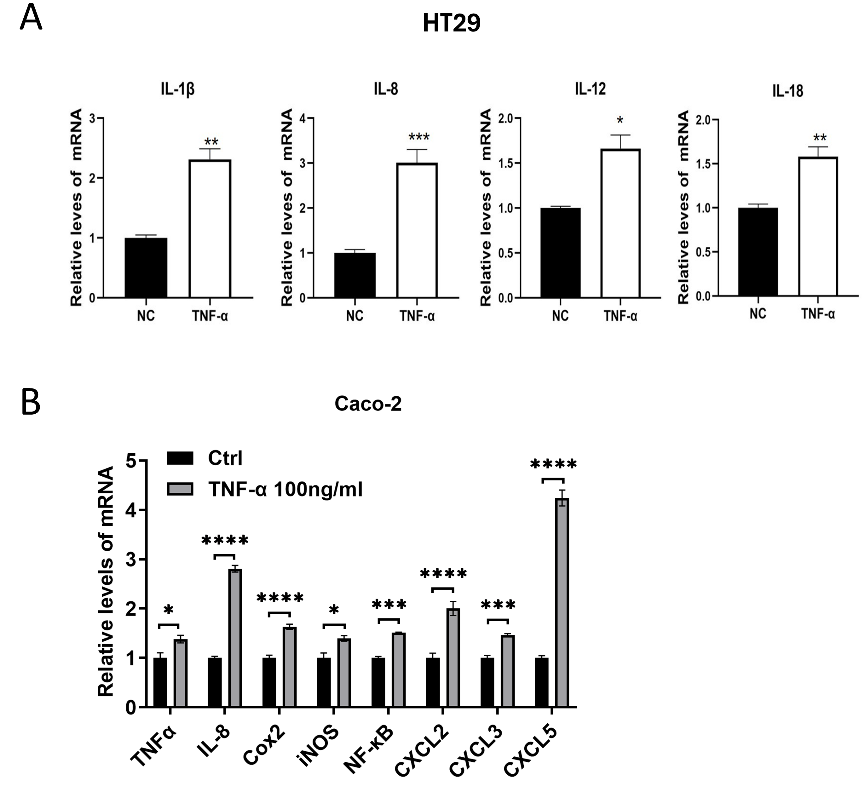


Suppl. Fig1 inflammatory cytokines were increased in vitro inflammatory model

(A) qPCR analysis of IL-1β, IL-8, IL-12 and IL-18 in NC and TNFα-stimulated HT-29 cells.

(B) qPCR analysis of TNFα, IL-8, Cox2, iNOS, NF-κB, CXCL2, CXCL3, CXCL5 in NC and TNFα-stimulated Caco-2 cells.

Suppl. Fig1 inflammatory were increased cytokines in vitro inflammatory model

(A) mRNA expression of inflammatory cytokines in the NC, NC+TNF-a groups in HT29 cells.

(B) mRNA expression of inflammatory cytokines in the NC, NC+TNF-a groups in Caco-2 cells.

Suppl. Fig1 inflammatory were increased cytokines in vitro inflammatory model

(A) mRNA expression of inflammatory cytokines in the NC, NC+TNF-a groups in HT29 cells.

(B) mRNA expression of inflammatory cytokines in the NC, NC+TNF-a groups in Caco-2 cells.
